# Transport kinetics of four- and six-coordinate platinum compounds in the multicell layer tumour model

**DOI:** 10.1038/sj.bjc.6603854

**Published:** 2007-06-19

**Authors:** S Modok, R Scott, R A Alderden, M D Hall, H R Mellor, S Bohic, T Roose, T W Hambley, R Callaghan

**Affiliations:** 1 Oxford Drug Resistance Group, Nuffield Department of Clinical Laboratory Sciences, University of Oxford, Level 4, The John Radcliffe Hospital, Oxford OX3 9DU, UK; 2 Centre for Heavy Metals Research, School of Chemistry, The University of Sydney, Sydney, NSW 2006, Australia; 3 ESRF, Micro-Fluorescence/Imaging/Diffraction – ID22, 6, rue Jules Horowitz, BP 220, F-38043 Grenoble Cedex, France; 4 Oxford Centre for Industrial and Applied Mathematics and Centre for Mathematical Biology, Mathematical Institute, University of Oxford, 24-29 St Giles', Oxford OX1 3LB, UK

**Keywords:** cisplatin, convection, diffusion, drug delivery, multicell layer, 3D tumour model

## Abstract

Four-coordinate (Pt(II)) platinum-based anticancer drugs are widely used in primary or palliative chemotherapy and produce considerable efficacy in certain clinical applications, for example testicular cancer. However, in many cancers the Pt(II) drugs are beset by poor efficacy mainly due to suboptimal pharmacokinetic properties. Consequently, the six-coordinate (Pt(IV)) class of Pt drugs were developed to improve platinum efficacy by (i) increasing stability, (ii) reducing reactivity, (iii) increasing lipophilicity, and (iv) nuclear targeting. However, comparatively little information is available on the pharmacokinetic properties of these compounds within solid tumour tissue. In the present study, the distribution and fluxes of [^14^C]-labelled [PtCl_2_(en)] (where en stands for ethane-1,2-diamine) and *cis,trans*-[PtCl_2_(OH)_2_(en)] drugs were determined in the multicell layer (MCL) tumour model comprising colon cancer cells. Flux data were analysed by mathematical modelling of drug diffusion and cellular uptake in the transport system. The flux of the Pt(IV) compound through the MCL was not significantly different to that of the Pt(II) drug nor were the diffusion coefficient or tissue uptake; the latter confirmed with elemental imaging analysis by synchrotron radiation induced X-ray emission. However, the flux of the Pt(IV) through the MCL was increased by hydrostatic pressure, thereby demonstrating the potential to target cancer cells further away from the vessels with six-coordinate platinum drugs.

Platinum drug complexes have been in clinical use for three decades and their greatest curative potential is exhibited towards certain subtypes of testicular cancer. The Pt complexes are widely used in cancer chemotherapy but unfortunately their efficacy is limited against the majority of malignancies. The reasons for the failure of this important class of chemotherapeutic agent are a combination of cellular drug resistance, toxicity and poor whole-body or cellular pharmacokinetic profiles (Wang and Lippard, 2005). Cellular resistance to Pt drugs is largely attributed to upregulation of DNA repair pathways, low intracellular accumulation and inactivation by thiol-containing reductants such as glutathione and metallothionein. Toxicity of cisplatin and its four-coordinate derivatives is due to inherently high reactivity, which leads to premature adduct formation with nucleophilic groups on macromolecules (Kartalou and Essigmann, 2001). The reactivity results from ‘aquation’ caused by the exchange of Cl^−^ moieties for OH^−^ in a low chloride environment, such as that in the intracellular milieu. The activation will ultimately manifest as the side-effect profile in addition to ensuring that only a minor fraction of the plasma platinum dose will be available to form adducts with the DNA (Lindauer and Holler, 1996). Platinum can exist in the oxidation states II and IV, and the presence of axial ligands in the six-coordinate Pt(IV) complexes has generated a series of compounds with considerably lower ‘aquation’ rates (Dolman *et al*, 2002). The resultant increased stability and reduced covalent binding to serum proteins (Dolman *et al*, 2002), facilitates delivery of Pt(IV) drug to cancer cells at effective concentrations.

The efficacy of Pt(II) complexes is severely limited due to poor cellular uptake. The main pathway for intracellular accumulation of Pt drugs is thought to be passive diffusion through the plasma membrane (Siddik, 2002), although the Ctr1 copper importer (Holzer *et al*, 2004) and the copper export pump ATP7B (Katano *et al*, 2004) have also been implicated. The introduction of hydrophobic axial ligands in Pt(IV) complexes was thought to provide a strategy to facilitate passive diffusion into cancer cells. Unfortunately, the correlation between lipophilicity and cellular uptake was weak for Pt(IV) compounds (Hall *et al*, 2004a), although among a range of Pt(IV) compounds *cis,trans*-[PtCl_2_(OH)_2_(en)] (where en stands for ethane-1,2-diamine) was the least lipophilic (log *P*_oct_: −2.78) and had the lowest cellular uptake. In contrast, there was a better correlation between the redox potential and uptake of these compounds. This finding has generated a model, which proposes that the Pt(IV) drug exists in a dynamic equilibrium across the cell membrane and that the preferable rate of intracellular ‘aquation’ and subsequent reactivity effectively traps the drug inside the cell (Hall *et al*, 2004b).

Despite the many advantageous features of Pt(IV) complexes that have been demonstrated in monolayer cultures of cancer cells, few of this class of drug have entered clinical trials. A possible explanation for this lack of translation may be the complexity of solid tumours *in vivo*, relative to the *in vitro* experimental system. Tumours *in vivo* represent complex three-dimensional cellular organisations with a characteristically harsh microenvironment comprising high cell density, low pH, toxic metabolites, low oxygenation and elevated interstitial pressure. Few studies have systematically examined the impact of these and other factors on the efficacy of Pt(IV) complexes in solid tumours or provided information on the distribution, diffusivity and flux of Pt(IV) complexes through a solid tumour. It is known that the driving force for passive diffusion through the plasma membrane is the concentration gradient of the platinum compound. Hence increased penetration of Pt(IV) complexes into tumour tissues, a higher local drug concentration and rapid intracellular aquation may significantly impact on the delivery of platinum compounds to nuclear DNA. Given the complexity of tumour architecture and biology there are many factors contributing to poor success; penetration and uptake are the first such factors that need to be overcome. Ensuring efficient delivery and uptake do not guarantee efficacy, but without them, drug activity may be limited.

This investigation has examined the pharmacokinetic properties of a reductively activated Pt(IV) compound in the multicell layer (MCL) three-dimensional tumour model. This model, unlike the more widely used tumour spheroid system, enabled a quantitative analysis of the distribution and flux parameters for Pt drugs (Hicks *et al*, 1997). The data verified a mathematical model developed to describe drug pharmacokinetics within solid tissue. Moreover, a novel elemental imaging analysis by synchrotron radiation induced X-ray emission (SRIXE) was applied to examine Pt distribution and the degree of intracellular accumulation in MCLs.

## MATERIALS AND METHODS

### Materials

**Figure 1:**
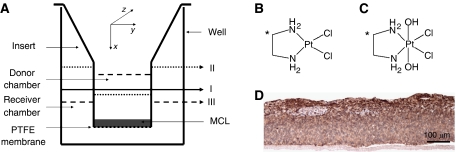
The MCL system and chemical structures of platinum compounds. (**A**) Transwel-Col inserts were placed with MCL grown on the PTFE membrane into the well of a 24-well tissue culture plate. The culture medium containing the radiolabelled compounds was added to the DC in variable fluid volumes (I–III) and fluxes were measured by taking samples from the RC. (**B** and **C**) The structures of the four (**B**; [^14^C]-*cis*-[PtCl_2_(en)]) and six-coordinate platinum compounds (**C**; [^14^C]-*cis*,*trans*-[PtCl_2_(OH)_2_(en)]). ^*^Position of the ^14^Carbon radioisotopes. (**D**) The DLD1 MCL were cultured in spinner flasks for 16 days and stained with the hypoxia probe pimonidazole on a 5 *μ*m paraffin cross-sections (× 10 objective) (for details see Materials and Methods).

Compounds [^14^C]-[PtCl_2_(en)] ([^14^C]-Pt(II); 34 Ci/mol) (Figure 1B) and [^14^C]-*cis,trans*-[PtCl_2_(OH)_2_(en)] ([^14^C]-Pt(IV); 6.8 Ci/mol) (Figure 1C) were synthesised according to published methods (Hall *et al*, 2004b). Platinum compounds were dissolved in 100 mM KCl in ddH_2_O at 0.5 mM concentration and stored in aliquots at −20°C. Twenty-four-well culture plates and Transwel-Col inserts (polystyrene sidewall, 6.5 mm internal diameter, bovine placental collagen type I- and III-coated polytetrafluoroethylene (PTFE) membrane, 0.33 cm^2^ surface, 400 nm average pore size) were obtained from Corning Life Sciences (Loughborough, UK), penicillin and streptomycin from Cambrex Bioscience (Verviers, France), spinner flasks were obtained from Techne Ltd (Cambridge, UK). All other cell culture materials were purchased from Invitrogen (Paisley, UK). Agarose was bought from BioWhittaker Biological Applications (Rockland, ME, USA) and Ready Protein^+^ scintillation liquid from Beckman Coulter Inc. (Fullerton, CA, USA). Hypoxyprobe™-1 kit was purchased from Chemicon Europe Ltd (Chandlers Ford, UK), Brij 35 from Fisher Scientific (Loughborough, UK) and peroxidase blocking agent from DAKO Corporation (Carpinteria, CA, USA). MACH2 horseradish peroxidase-labelled anti-mouse secondary antibody was obtained from Biocare Medical (Concord, CA, USA) and DAB substrate chromogen from DakoCytomation (Ely, UK). Haematoxylin and Aquamount were purchased from BDH Laboratory Supplies (Lutterworth, UK). All other chemicals were from Sigma and at least analytical grade.

### Cell lines and MCL culture

DLD1 colon carcinoma cells were a kind gift of Dr Roger Phillips (Bradford, UK) and were maintained according to published protocols (Hall *et al*, 2004b). DLD1 cell monolayers were detached from culture flasks with trypsin/EDTA (Invitrogen) and 3 × 10^5^ cells were seeded into Transwel-Col inserts. After a few hours the cells settled and the inserts were transferred into spinner flasks (Techne Ltd) with 50 ml RPMI 1640 medium per insert and half of the medium was refreshed every third day.

### Measuring flux of radiolabelled compounds through the MCL system

Triplicate inserts, with or without MCLs, were transferred into 24-well plates with 1000 *μ*l fresh medium in the wells (Figure 1A). At the beginning of the transport assay the medium in the donor compartment (DC) was replaced with fresh RPMI 1640 medium containing the [^14^C]-labelled platinum compound. The volume in the DC was 150 and 1000 *μ*l in the RC. Under these conditions, the fluid levels between the receiver compartment (RC) and DC were level and consequently, transport was driven by the concentration gradient. In subsequent experiments, to provide convective force against the concentration gradient-driven diffusion, the volume of the RC was increased to 1500 *μ*l. The reverse gradient was created by increasing the DC volume to 280 *μ*l and keeping the RC at 1000 *μ*l. These two configurations provide ±3 mm H_2_O hydrostatic pressure between the two compartments. The RC was not stirred because the flux rates across the membrane were identical with or without stirring (data not shown). The experiment apparatus was kept in the incubator (37°C) and at each time point the insert was moved to a new well. An 800 *μ*l sample was taken from the RC and the radioactivity at each time point was measured in a Beckman liquid scintillation counter after mixing the sample with 4 ml Ready Protein^+^ scintillation liquid.

Non-specific binding of radiolabelled compounds to the transport apparatus can influence the interpretation of transport results. Consequently, non-specific binding to polystyrene surfaces in the RC was determined by measuring the decrease in drug concentration within the compartment following 2 h incubation with [^14^C]-Pt complex. The [^14^C]-Pt complex was added as a 9 : 1 mixture of non-labelled and radiolabelled complexes in culture medium at the concentration used during the transport assays. The cumulative amount of drug in the RC was corrected for non-specific binding to the RC.

A similar correction was made for drug concentrations in the DC. After completion of transport assays, samples were taken from the DC and the PTFE membranes with the MCL were excised to measure bound radioactivity. In control experiments without MCLs the PFTE membrane was excised. The percentage of added [^14^C]-Pt complex bound to the DC was determined by subtracting the percentage bound to PTFE membrane in the presence or absence of the MCL, the percentage transported to the RC and from the percentage decrease in the DC at the end of the transport assay.

### SRIXE of MCL cross-sections

MCLs were harvested at the end of the transport experiment using the Pt(IV) compound, processed as described (Modok *et al*, 2006), and 20 *μ*m sections were cut from the paraffin blocks. The sections were mounted on Formvar-coated plastic specimen holders. Micro-SRIXE experiments were performed on beamline ID22 at the European Synchrotron Radiation Facility (ESRF; Grenoble, France). Fluorescence spectra were collected using a single element Si(Li) detector, placed approximately 20 mm from the sample. Two-dimensional maps corresponding to the integrated *K*α fluorescence signal of an element of interest were collected by scanning the sample. The elements analysed were P, S, Cl, K, Ca, Fe, Ni, Cu, Zn, and Pt which was analysed using the L*α* and L*β* fluorescence lines. Elemental contents were quantified by comparison to the SRM 1832 and 1833 thin polymer film standards (NBS/NIST, Gaithersburg, MD, USA) using the assumption for a thin film target. Sectioned MCLs were analysed using a 13 keV monochromatic X-ray beam focused to a 1.8 *μ*m × 4.5 *μ*m (vertical × horizontal) spot. Samples were mounted and suitable areas of the MCL located by viewing the sample with a video-zoom microscope. Scan areas were chosen so as to incorporate a representative cross-section of the MCL. PyMca, an ESRF code, was used for fitting the acquired spectra (http://www.esrf.fr/computing/bliss/downloads/pymca/PyMCA.pdf). This allowed the background contribution to be removed, facilitated analysis of overlapping peaks, and accounted for the eventual escape peaks. X-ray lines were fitted using the Hypermet function and a model for background fitting was chosen as a tenth order polynomial for an exponential background model. Computed X-ray line intensities were normalised to the value of the incident photon flux.

Data analysis was performed by choosing regions of interest (ROIs) within each scan. Such ROIs typically included one ROI encompassing the majority of the scan area (‘whole MCL’), another encompassing the free surface of the tissue (i.e. DC surface), and another encompassing part of the PTFE membrane (i.e. RC surface). The average fluorescence spectra from each ROI were fitted using the PyMca X-ray fluorescence fitting program. Elemental concentrations (*μ*g cm^−2^) were calculated from peak areas using peak area:concentration ratios determined from NIST thin-film standards (SRM 1832 and 1833), and then converted to *μ*g cm^−3^ by accounting for the known section thicknesses. Such calculations neglected secondary fluorescence processes and assumed that the standards and the sample were similar to one another. The Pt-L*α* fluorescence line was used to quantify platinum content in a similar fashion to that outlined by Ilinski *et al* (2003) using fluorescence line cross-sections (at 13 keV) of Zn-*Kα*=43.089 cm^2^g^−1^, and Pt-L*α*=18.208 cm^2^g^−1^ (Brunetti *et al*, 2004). Elemental content of ROIs were compared to that of the ‘whole MCL’ ROI, and the zinc content of the ROI.

### Detection of hypoxia in MCL

Multicell layers were incubated with 200 *μ*M pimonidazole (Hypoxyprobe-1) in culture medium for 2 h and then fixed in 4% buffered paraformaldehyde. The fixed MCLs were extracted and embedded in 4% agarose as described previously (Modok *et al*, 2006). Multicell layers in agarose moulds were dehydrated through routine histological processing and embedded in paraffin for subsequent sectioning (5 *μ*m). Sections were de-waxed and the pimonidazole detected with the Hydroxyprobe-1 monoclonal antibody and the MACH2 secondary antibody as described previously (Mellor *et al*, 2005).

### Mathematical model to describe intra-MCL drug pharmacokinetics

*Model to describe drug flux through Transwel-Col membranes* The experimental system is depicted in Figure 1A and is modelled as a closed system with mass conservation: 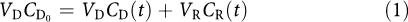 where *V*_D_ and *V*_R_ are the volumes of the DC and RC (ml), respectively, and *C*_Do_ is the initial concentration of the radiolabelled compound in the donor chamber (nM), whereas C_B_(0)=0 nM. The flux through the membrane is proportional to the membrane surface area (*A*, cm^2^) and the concentration gradient across the membrane. Furthermore, the flux through the membrane equals the change of concentration of molecules in the RC, hence we get 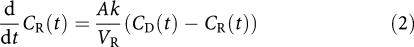 where *k* is the mass transfer coefficient (cm s^−1^). *C*_D_ can be eliminated using Eq. 1 and then Eq. 2 solved to give 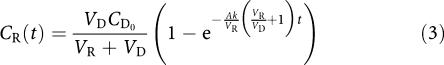 The fitted parameter was *k* and the relative porosity of the membrane for the compounds (∼impedance, *ψ*) was determined according to 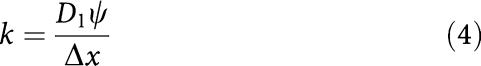 where *D*_1_ is the diffusion coefficient of the compound in medium (cm^2^ s^−1^) and Δ*x* is the measured thickness of the membrane (cm).

*Mathematical model to describe flux of drugs through the MCL* The transport of radiolabelled compounds through the MCL was modelled as Fickian diffusion. The insert with the MCL can be seen as an anisotropic cylinder which has its axis along the direction *x* (Figure 1A) and is bounded by planes perpendicular to *x* and the problem of diffusion into it reduces to the corresponding problem in an isotropic cylinder provided *D*_y_=*D*_z_, where *D*_y_ and *D*_z_ are diffusion constants in the other two directions of space (Dolman *et al*, 2002). As the concentration only varies along the *x* axis, we can describe the diffusion of the drug in the MCL by 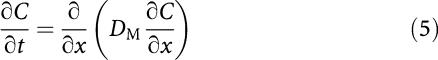 where *D*_M_ is the diffusion coefficient of the compound in the MCL (cm^2^ s^−1^) and *x* is the distance from the top free surface of the MCL (cm), and *g* (s^−1^) is the rate of cellular uptake of the compound. *D*_M_ can be defined by the impedance of MCL (*Γ*) and the diffusivity of the compound in medium (*D*_1_, cm^2^ s^−1^) as 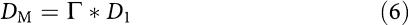 The boundary conditions were at *x* = 0*C*(0,*t*) = *C*_D_(*t*), where the concentration in top compartment is calculated from the mass balance by considering the flux of molecules that enters the MCL, i.e., 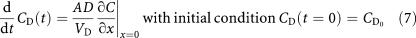 The continuity of the flux at the interface between the MCL and the membrane gives us another boundary condition at 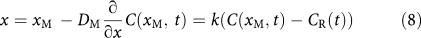 where *x*_M_ is the thickness of the MCL. Furthermore, the governing equation for the drug concentration in the RC (*C*_R_(*t*)) is at 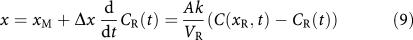

Eqs. 5, 6, 7, 8 and 9 are coupled partial differential equations, which were used to write a program in Matlab to simulate the experimental situation with initial conditions *C*=0 at 0<*x*⩽*x*_M_ and *C*_R_(0)=0 to calculate *Γ*, *D*_M_ and *g*.

### Data analysis

Data were presented as either mean±s.d. or mean±s.e.m. The data were analysed with two-tailed *t*-test using GraphPad Prism 3.2 software and *P*<0.05 was considered statistically significant. Diffusivity and the rate of cellular uptake were determined using programs based on the mathematical models described above and written in Matlab 7.0.1 software.

## RESULTS

### Growth and histological characterisation of MCL

Multicell layers were cultured from DLD1 colon adenocarcinoma cells on collagen-coated PTFE membranes (Figure 1), which were floated in spinner flasks to provide optimised conditions for growth. The MCL were used on the seventh day of culture and displayed a tissue depth of 131±10 *μ*m. A pimonidazole-based assay was used to detect hypoxia in the MCL and demonstrate tissue morphology (Figure 1D). Full morphological characterisation of this tissue model was described previously (Modok *et al*, 2006). The MCL showed pimonidazole staining towards the upper surface of the tissue, which is indicative of hypoxia. This localised hypoxia may be explained by the high demand for oxygen, which results from such a densely packed avascular population of cells, akin to that in tumour spheroids. This suggests that the MCL model is a good representation of solid tumour architecture and the associated microenvironment (Modok *et al*, 2006).

### Diffusivity and cellular uptake rate of [^14^C]-Pt(II) and [^14^C]-Pt(IV) compounds in MCL

The transport of the compounds through the PTFE membrane with or without the MCL was measured by loading the radiolabelled drugs to the DC and measuring the radioactivity appearing in the RC over time (Figure 1A). Initially, 150 and 1000 *μ*l volumes of culture medium were loaded to the DC and RC respectively, to maintain an equivalent fluid level and therefore avoid hydrostatic pressure-driven convective transport. The non-specific binding of the compounds to the plastic surfaces in DC and RC was determined to improve the accuracy of the subsequent quantitative analyses (Table 1). For example, the fraction bound to the DC reduces the effective concentration gradient, whereas the fraction bound to the RC falsely reduces the flux rate. The fractions bound to the DC and to the RC were used to correct the starting drug concentration and the concentration of the drug transported, respectively. The cumulative concentration of [^14^C]-Pt(II) and [^14^C]-Pt(IV) were expressed as a percentage of the initial drug concentration in the DC.

**Figure 2:**
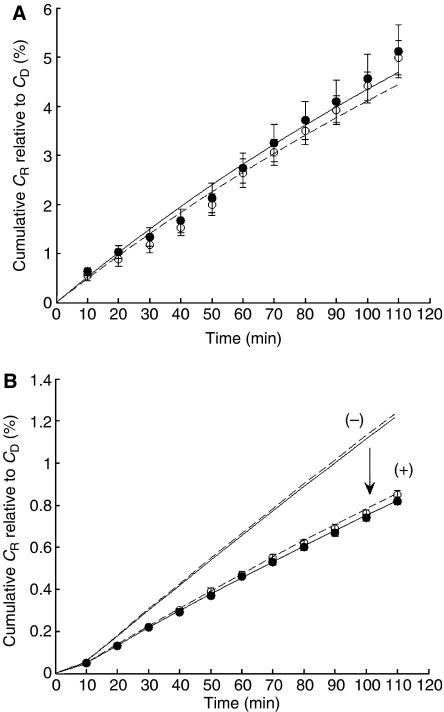
Flux kinetics of [^14^C]-Pt(II) and [^14^C]-Pt(IV) through MCL. Typically, concentrations of 6 *μ*M [^14^C]-Pt(II) (•) and 18 *μ*M [^14^C]-Pt(IV) (○) were administered to the DC. Appearance of radiolabelled compound in the RC was measured as described in Materials and Methods. The cumulative concentration in the CR (*C*_R_) was divided by the starting concentration in the DC (*C*_D_) and plotted as the mean±s.d. percentage of three independent experiments. The curves were fitted using the mathematical models with (+) or without (−) terms for cellular uptake as described in the Materials and Methods. Flux was measured through (**A**) the PFTE membrane only and (**B**) MCL grown on the PFTE membrane.

Figure 2 shows the cumulative amounts of Pt complexes appearing in the DC of the MCL apparatus. Data are provided for fluxes in the presence or absence of the MCL tissue overlaid on the PTFE membrane. As expected, the rate and extent of flux was greater for both drugs in the absence of the MCL. The data in Figure 2 clearly demonstrates that there was no difference between [^14^C]-Pt(IV) and [^14^C]-Pt(II) with respect to their cumulative appearance in the DC. Mathematical modelling without a cellular uptake parameter did not fit the data obtained in the presence of an MCL, but was an accurate reflection for passage across the PTFE membrane alone. The mathematical model comprising an uptake coefficient was used to quantify flux of the compounds through the solid tumour model and the data are summarised in Table 2. The mass transfer coefficients (*k*) and relative porosity (*ψ*) values of the two platinum compounds through MCLs were indistinguishable. The coefficient of diffusion was ascertained for the Pt(IV) and Pt(II) drugs through the MCL model utilising the parameters of mass transfer coefficient (*k*), MCL thickness (*x*_M_), diffusivity in the medium (*D*_1_) and initial drug concentration (*C*_D_). The calculated diffusion coefficients (*D*_M_) and cellular uptake (*g*) rates were not different for the two platinum compounds (Table 2), reflecting similar flux parameters for the two classes of platinum complex in a solid tissue.

### Cellular accumulation of [^14^C]-Pt in the MCL; SRIXE analysis

**Figure 3:**
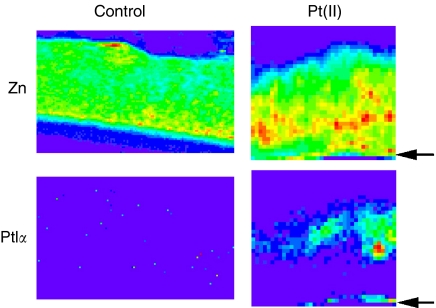
The cellular accumulation of [^14^C]-Pt(IV) in MCL: SRIXE analysis. After the transport assay the MCL was processed, embedded in wax and 20 *μ*m sections through the MCL were imaged in a synchrotron x-ray beam using SRIXE. Fitted images of the elemental distributions in segments of a Pt(IV)-treated MCL and a control non-treated MCL are shown. The scale on the axes represents the number of pixels, where each pixel is 3 × 2 *μ*m (horizontal x vertical). The images show relative elemental concentrations, using the colour scale shown, which ranges from blue, representing low levels, to red, representing high elemental levels. The arrowheads indicate the position of the PTFE membrane supporting the MCL (shown in Figure 1D).

**Figure 4:**
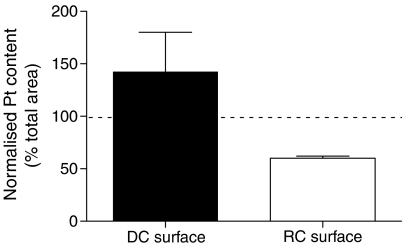
Quantification of the cellular accumulation of [^14^C]-Pt(IV) in MCL. The platinum contents of the surfaces exposed to the DC and the RC were quantified and normalised relative to a region representative of the entire MCL. Values are expressed as percentages, where a value of 100 is indicative of an elemental content identical to that of the whole MCL. The data represent the mean and s.e. associated with two scans.

Accumulation of Pt compounds in the MCL was determined by measuring the radioactivity retained in the PTFE membrane±MCL after the transport assays. As shown in Table 1, the radioactivity associated with the membrane was higher in the presence of the MCL, although there was no significant difference between [^14^C]-Pt(II) compared to [^14^C]-Pt(IV). Data from the mathematical modelling above suggested a significant degree of uptake for Pt drugs within the MCL tissue. Image analysis using SRIXE was used to confirm this suggestion and the technique enabled an extensive elemental analysis of the MCL tissue. The data show that the tissue that had been exposed to the [^14^C]-Pt(IV) drug was associated with an incorporated Pt signal (Figure 3). This confirmed the experimental and mathematical modelling data and demonstrates that flux through solid tissue is associated with a non-negligible degree of Pt drug uptake into cells. Quantitation of the Pt signal across the tissue revealed a greater Pt(IV) complex concentration at the upper surface (i.e. facing the DC) (Figure 4).

### The effect of hydrostatic pressure on the flux of [^14^C]-Pt(IV) through MCL

**Figure 5:**
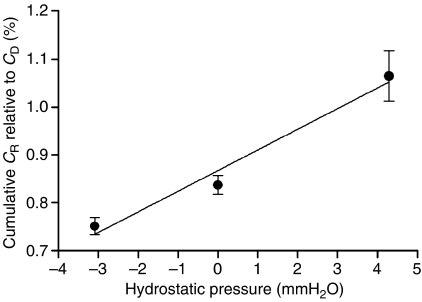
The effect of hydrostatic pressure on flux kinetics in MCL. Hydrostatic pressure was applied by varying the volume of medium added to the DC and the RC as described in Materials and Methods. The flux kinetics [^14^C]-Pt(IV) were determined and the cumulative RC concentration relative to the diffusion only values are shown as mean±s.e.m. of at least three independent experiments. ^*^Statistically significant difference (*P*<0.05).

The six-coordinate Pt(IV) compounds have been proposed to display a greater likelihood of reduction to the active Pt(II) species in the hostile intratumour microenvironment. Consequently, ensuring sufficient penetration of Pt(IV) drugs to the deeper layers of a tumour remains a key issue. One proposed way of improving penetration is to increase the hydrostatic pressure to counteract the increased interstitial pressure generated in tumours. The MCL system (Figure 1) provides a convenient means to investigate whether hydrostatic pressure can modulate the flux of [^14^C]-Pt(IV) through solid tumour tissue. Hydrostatic pressure may be varied by simply altering the fluid levels in the DC and RC and was applied in both the same and opposite directions to the concentration gradient of the MCL system. Figure 5 shows the cumulative amount of [^14^C]-Pt(IV) that had penetrated through the MCL in to the lower RC, relative to the amount of drug added originally to the DC. At a hydrostatic pressure of −3 mm H_2_O (i.e. against concentration gradient), the amount of penetration was reduced from 0.82±0.01 to 0.73±0.01%. In contrast, as the hydrostatic pressure was increased to +4 mm H_2_O (i.e. along the concentration gradient), the flux of [^14^C]-Pt(IV) was increased to 1.06±0.08%. The data therefore indicate that even a relatively minor hydrostatic pressure gradient was capable of a significant influence on the flux of [^14^C]-Pt(IV) through the MCL tumour model.

## DISCUSSION

Platinum-based chemotherapy remains a vital component of many oncological treatment strategies. As with the majority of ‘genotoxic’ anticancer drugs, the Pt complexes are beset by problems with toxicity, resistance and poor pharmacokinetic properties. A great deal of effort has been placed in generating more potent and selective derivatives of conventional Pt(II) drugs such as cisplatin. In particular, the six-coordinate Pt(IV) complexes offer several advantages including lower reactivity and a greater potential for introducing hydrophobicity to facilitate cellular uptake. There is little information available on either class of Pt drug relating to their behaviour in solid tumour tissue. This study has utilised a solid tumour model to quantify flux behaviour of Pt(II) and Pt(IV) complexes. Flux through the solid tumour model was associated with significant cellular accumulation and was sensitive to changes in the applied hydrostatic pressure.

The MCL system is widely accepted as a good model of solid tumour tissue, in particular the non-vascularised regions or nodules. The colon cancer MCLs described previously (Modok *et al*, 2006), display heterogeneity of cell status (i.e. proliferating and quiescent) in addition to having a high cell density and localised regions of hypoxia. Both of the compounds tested were able to completely penetrate the MCL tissue with the diffusion coefficients of 17.5±2.6 × 10^−8^ cm^2^ s^−1^ for the Pt(II) and 17.8±3.1 × 10^−8^ cm^2^s^−1^ for the Pt(IV) complex. These rates of diffusion were almost 10-fold higher than that reported for the hydrophobic anticancer drug vinblastine (1.88±0.21 × 10^−8^ cm^2^ s^−1^) and fourfold higher than that of the highly hydrophilic sucrose (4.22±0.89 × 10^−8^ cm^2^ s^−1^) (Modok *et al*, 2006). A similar rapid flux (i.e. % drug min^−1^) of cisplatin compared to vinblastine has been reported in MCL comprising bladder cancer cells, whereas etoposide, gemcitabine and paclitaxel displayed faster rates of flux (Tannock *et al*, 2002). A major advantage of the current approach is the ability to calculate the actual diffusion coefficients, which may be directly applied to the prediction of tissue drug concentrations; for example, as a function of the distance from a blood vessel (Modok *et al*, 2006). This rapid flux across the MCL should be considered a positive feature for such compounds. A considerable emphasis in the design of platinum drugs has been placed on developing lipophilic drugs that can assist in circumventing diminished cisplatin accumulation in resistant cell lines; however, it may be that defeating unicellular resistance may come at the expense of tissue penetration, and therefore create the problem of multicellular resistance. It seems that hydrophilic drugs such as those examined here allow for excellent tissue penetration. If this feature can be coupled with a more readily reducible Pt(IV) compound (the Pt(IV) compound studied here is the most inert in a series studied), a drug with good penetration and cytotoxic potential may be arrived at.

The Pt(IV) compound used in this study has been shown to display lower cellular accumulation in well-oxygenated monolayers of cancer cells (Hall *et al*, 2004a) and lower serum protein binding compared to the Pt(II) parental derivative (Dolman *et al*, 2002). Therefore Pt(IV) would be expected to display higher flux rates across MCL than Pt(II); unless the compound is rapidly converted into the parental Pt(II) version. The latter is highly unlikely given that in cells treated with platinum(IV) complexes, 50% of a *trans*-Pt(IV) dihydroxo complex remained oxidised 2 h after incubation, which corresponds to the longest time point in these experiments (Hall *et al*, 2003). The two Pt compounds had undistinguishable flux kinetics across the MCL and in both cases the mathematical model required the inclusion of a specific component to account for cellular uptake within the tissue. Similarly, the calculated diffusion constants and cellular uptake rates also failed to detect any pharmacokinetic differences between the two compounds. What is the explanation for the similar rates of flux through the solid tissue given the physico-chemical differences between Pt(II) and Pt(IV) species? Certainly neither compound has reacted extensively with growth media in the time examined. Perhaps the cellular accumulation of [^14^C]-Pt(IV) is limited by the lack of reactivity, allowing passage through the MCL at a rate similar to the more lipophilic Pt(II) complex. If the Pt(IV) compound was more reactive and thus converted into the corresponding Pt(II) compound, the concentration gradient into the cell for the Pt(IV) would remain high enough to ensure greater uptake. Consequently, future Pt(IV) development would require taking into account both transbilayer diffusion and the intracellular reactivity.

In addition to allowing detailed flux analysis, the mathematical model predicts that a concentration gradient would exist within the MCL decreasing from the free surface of the tissue towards the semiporous PTFE membrane (i.e. DC → RC direction) (Modok *et al*, 2006). The SRIXE element array qualitatively demonstrated that the distribution of the Pt(IV) compound was similar to that predicted by the mathematical model, that is, more Pt was detected close to the free surface of the MCL. This is the first report where the distribution of drugs within the MCL was experimentally verified and compared to the one predicted by mathematical modelling. That more Pt was observed close to the upper surface of the MCL is in keeping with previous observations with cisplatin detected by antibody in squamous carcinoma cell spheroids, and a Pt-porphyrin compound in J82 spheroids observed by fluorescence microscopy, which inferred more intense drug signal (although both relied on a molecular signature other than the active Pt centre itself) at the periphery of spheroids (Nishikawa *et al*, 1990; Lottner *et al*, 2004). In the case of the Pt-porphyrin complex, it took 24 h before uniform drug distribution could be achieved, although this is not reflective of the drug distribution expected to be achieved by a pulse of chemotherapeutic agent.

Most solid tumours have high interstitial pressures that slow drug transit and filtration through tissue and consequently drug movement in cancer tissue is largely limited to diffusion (Heldin *et al*, 2004). Restoring the physiological filtration by increasing the hydrostatic pressure is a possible option to improve drug delivery to deeper layers of cancer cells within the three-dimensional solid tumour mass. Our results show that because the flux of the two compounds was sensitive to hydrostatic pressure the convection could potentially increase the penetration of tumour mass by platinum drugs. This principle, known as convection enhanced delivery (CED), has actually been utilised to treat intracranial tumours where the confined space further increases the effects of interstitial pressure (Heldin *et al*, 2004). Cisplatin is expected to be useful via local drug delivery (Newton, 2005) in intracranial tumours due to its reported rapid flux rate, although an enhancement by CED has not yet been examined.

In conclusion, the data from this investigation provide hitherto unknown pharmacokinetic properties for two platinum compounds; namely the absolute diffusion coefficient through solid tumour tissue. Moreover, both experimental and mathematical models provide a convenient means to facilitate the *in vitro* development of novel six-coordinate platinum compounds; particularly to enable a bridge between observations in simple cell monolayer systems to the complexities associated with poorly vascularised hypoxic tumours *in vivo*.

## Figures and Tables

**Table 1:** Binding of the radiolabelled compounds in the MCL system

	**Membrane**		**MCL**	
**%, mean±s.d.**	**[^14^C]-Pt(II)**	**[^14^C]-Pt(IV)**	**[^14^C]-Pt(II)**	**[^14^C]-Pt(IV)**
Added to DC	100	100	100	100
Decrease in DC^a^	46.3±2.6, *n*=3	43.06±2.6, *n*=3	17.6±3.6, *n*=12	15.3±4.1, *n*=12
Membrane±MCL^b^	0.1±0.1, *n*=3	0.3±0.1, *n*=5	0.7±0.3^*^, *n*=6	0.5±0.2^*^, *n*=9
				
Transported^c^	27.8±5.0, *n*=3	28.6±3.5, *n*=3	5.3±0.8, *n*=12	5.3±0.5, *n*=12
				
Bound to RC^d^	12.8±0.4, *n*=3	16.6±1.2, *n*=3	12.8±0.4, *n*=3	16.6±1.2, *n*=3
				
Bound to DC^e^	18.5±7.0, *n*=3	13.9±5.6, *n*=3	10.1±2.8, *n*=6	10.5±4.3, *n*=9

Abbreviations: DC, donor chamber; en, ethane-1,2-diamine; MCL, multicell layer; [^14^C]-Pt(II), [^14^C]-[PtCl_2_(en)]; [^14^C]-Pt(IV), [^14^C]-*cis,trans*-[PtCl_2_(OH)_2_(en)].

The percentage bound to the DC^e^ was calculated by subtracting the percentage bound to membrane±MCL^b^ and the percentage transported to the receiver chamber RC^c^ from the percentage decrease in the DC^a^ at the end of the transport assay. These values and the non-specific binding of radiolabelled compounds to the RC^d^ were measured as it is described in Materials and Methods. ^*^Statistically significant (*P*<0.5) accumulation of the Pt compounds in the MCL compared to the PTFE membrane alone.

**Table 2:** Physical and chemical parameters of radiolabelled platinum compounds

	**[^14^C]-Pt(II)**	**[^14^C]-Pt(IV)**
Molecular weight	325	359
		
Molecular radius (nm)	0.4^a^	0.4^a^
Diffusion coefficient in medium (*D*_1_, × 10^−6^ cm^2^ s^−1^)	8.2^b^	8.2^b^
Mass transfer coefficient (*k*, × 10^−5^ cm s^−1^)	2.7±0.6, *n*=3	2.5±0.4, *n*=3
Relative porosity of membrane (*ψ*; × 10^−2^)	1.2±0.3, *n*=3	1.2±0.2, *n*=3
Impedance of MCL (*Γ*; ^*^10^−2^)	2.1±0.3, *n*=3	2.2±0.4, *n*=4
Diffusion coefficient in MCL (*D*_M_; × 10^−8 ^cm^2^ s^−1^)	17.5±2.6, *n*=3	17.8±3.1, *n*=4
Uptake rate in MCL, (*g*; × 10^−2^ min^−1^)	17.7±5.5, *n*=3	16.2±5.3, *n*=4

a The molecular radius of Pt(II) and Pt(IV) was estimated based on the size of carboplatin (8 × 4 × 3 Å) and cisplatin (5 × 3 × 1.5 Å) as measured in MDLChime.

b The diffusion coefficient of platinum compounds in culture medium was calculated according to the Stokes–Einstein equation using the molecular radius.

## References

[bib1] Brunetti A, del Rio MS, Golosio B, Simionovici A, Somogyi A (2004) A library for X-ray matter interaction cross sections for X-ray fluorescence applications. Spectrochim Acta B 59: 1725–1731

[bib2] Dolman RC, Deacon GB, Hambley TW (2002) Studies of the binding of a series of platinum(IV) complexes to plasma proteins. J Inorg Biochem 88: 260–267 11897339 10.1016/s0162-0134(01)00360-9

[bib3] Hall MD, Amjadi S, Zhang M, Beale PJ, Hambley TW (2004a) The mechanism of action of platinum(IV) complexes in ovarian cancer cell lines. J Inorg Biochem 98: 1614–1624 15458824 10.1016/j.jinorgbio.2004.05.017

[bib4] Hall MD, Foran GJ, Zhang M, Beale PJ, Hambley TW (2003) XANES determination of the platinum oxidation state distribution in cancer cells treated with platinum(IV) anticancer agents. J Am Chem Soc 125: 7524–7525 12812486 10.1021/ja0354770

[bib5] Hall MD, Martin C, Ferguson DJP, Phillips RM, Hambley TW, Callaghan R (2004b) Comparative efficacy of novel platinum(IV) compounds with established chemotherapeutic drugs in solid tumour models. Biochem Pharmacol 67: 17–30 14667925 10.1016/j.bcp.2003.07.016

[bib6] Heldin CH, Rubin K, Pietras K, Ostman A (2004) High interstitial fluid pressure – an obstacle in cancer therapy. Nat Rev Cancer 4: 806–813 15510161 10.1038/nrc1456

[bib7] Hicks KO, Ohms SJ, van Zijl PL, Denny WA, Hunter PJ, Wilson WR (1997) An experimental and mathematical model for the extravascular transport of a DNA intercalator in tumours. Br J Cancer 76: 894–903 9328149 10.1038/bjc.1997.481 PMC2228074

[bib8] Holzer AK, Katano K, Klomp LWJ, Howell SB (2004) Cisplatin Rapidly Down-regulates Its Own Influx Transporter hCTR1 in Cultured Human Ovarian Carcinoma Cells. Clin Cancer Res 10: 6744–6749 15475465 10.1158/1078-0432.CCR-04-0748

[bib9] Ilinski P, Lai B, Cai Z, Yun W, Legnini D, Talarico T, Cholewa M, Webster LK, Deacon GB, Rainone S, Phillips DR, Stampfl AP (2003) The direct mapping of the uptake of platinum anticancer agents in individual human ovarian adenocarcinoma cells using a hard X-ray microprobe. Cancer Res 63: 1776–1779 12702562

[bib10] Kartalou M, Essigmann JM (2001) Recognition of cisplatin adducts by cellular proteins. Mutat Res 478: 1–21 11406166 10.1016/s0027-5107(01)00142-7

[bib11] Katano K, Safaei R, Samimi G, Holzer A, Tomioka M, Goodman M, Howell SB (2004) Confocal Microscopic Analysis of the Interaction between Cisplatin and the Copper Transporter ATP7B in Human Ovarian Carcinoma Cells. Clin Cancer Res 10: 4578–4588 15240550 10.1158/1078-0432.CCR-03-0689

[bib12] Lindauer E, Holler E (1996) Cellular distribution and cellular reactivity of platinum (II) complexes. Biochem Pharmacol 52: 7–14 8678910 10.1016/0006-2952(96)00106-2

[bib13] Lottner C, Knuechel R, Bernhardt G, Brunner H (2004) Distribution and subcellular localization of a water-soluble hematoporphyrin-platinum(II) complex in human bladder cancer cells. Cancer Lett 215: 167–177 15488635 10.1016/j.canlet.2004.06.035

[bib14] Mellor HR, Snelling S, Hall MD, Modok S, Jaffar M, Hambley TW, Callaghan R (2005) The influence of tumour microenvironmental factors on the efficacy of cisplatin and novel platinum(IV) complexes. Biochem Pharmacol 70: 1137–1146 16139250 10.1016/j.bcp.2005.07.016

[bib15] Modok S, Hyde P, Mellor HR, Roose T, Callaghan R (2006) Diffusivity and distribution of vinblastine in three-dimensional tumour tissue: Experimental and mathematical modelling. Eur J Cancer 42: 2404–2413 16901688 10.1016/j.ejca.2006.05.020

[bib16] Newton HB (2005) Intra-arterial chemotherapy of primary brain tumors. Curr Treat Options Oncol 6: 519–530 16242056 10.1007/s11864-005-0030-1

[bib17] Nishikawa K, Newman RA, Murray L, Khokhar AR, Rosenblum MG (1990) Detection of cellular platinum using the monoclonal antibody 1C1. Mol Biother 2: 235–241 2288724

[bib18] Siddik ZH (2002) Biochemical and molecular mechanisms of cisplatin resistance. Cancer Treat Res 112: 263–284 12481720 10.1007/978-1-4615-1173-1_13

[bib19] Tannock IF, Lee CM, Tunggal JK, Cowan DS, Egorin MJ (2002) Limited penetration of anticancer drugs through tumor tissue: a potential cause of resistance of solid tumors to chemotherapy. Clin Cancer Res 8: 878–884 11895922

[bib20] Wang D, Lippard SJ (2005) Cellular processing of platinum anticancer drugs. Nat Rev Drug Discov 4: 307–320 15789122 10.1038/nrd1691

